# Ethyl pyruvate treatment mitigates oxidative stress damage in cultured trabecular meshwork cells

**Published:** 2013-06-11

**Authors:** Amin Famili, David A. Ammar, Malik Y. Kahook

**Affiliations:** 1 Department of Bioengineering, University of Colorado Denver, Aurora, CO; 2 Department of Ophthalmology, University of Colorado Denver, MS F731 Rocky Mountain Lions Eye Institute, Aurora, CO

## Abstract

**Purpose:**

Oxidative stress plays a key role in the pathophysiology of glaucoma. This study was designed to assess ethyl pyruvate (EP) as a novel antioxidative agent in cultured human trabecular meshwork (hTM) cells.

**Methods:**

Primary hTM cells were cultured on collagen matrices. Tolerance to EP was assessed at various concentrations using fluorescent vital dyes (live/dead) and metabolic (1-(4,5-dimethylthiazol-2-yl)-2,5-diphenyltetrazolium bromide) assays. After the candidate doses were identified, cells received either preincubation with EP before hydrogen peroxide stressing or pre- and coincubation with EP before and during stressing. Live/dead and metabolic activity assays were used to quantify oxidative damage.

**Results:**

Cultured hTM cells were well tolerant of EP concentrations at or below 10 mM while higher doses showed significant levels of cytotoxicity. In the peroxide stress assays, samples that received pre- and cotreatment with all concentrations of EP showed significantly increased cell survival and maintenance of metabolic activity. However, samples that received only pretreatment did not show a significant increase in survival rates and lost nearly all metabolic activity after peroxide-induced stressing.

**Conclusions:**

This work suggests that EP is a potent antioxidant that is well tolerated by hTM cells; however, EP’s potential as a therapeutic agent for glaucoma is limited by its inability to enhance endogenous antioxidant capacity. A continuous drug delivery system may be needed to realize the full therapeutic potential of EP for treatment of glaucoma.

## Introduction

Oxidative stress has been implicated as a key step in the progression of various ocular pathologies including age-related macular degeneration (AMD) [1,2], cataract [3,4], and glaucoma [5-7]. Dysregulation of outflow facility [8] and retinal ganglion cell (RGC) apoptosis [5,9,10], both important components of the pathophysiology of glaucoma, have been connected to oxidative stress. Morphological and biochemical changes in the trabecular meshwork (TM) of patients with primary open-angle glaucoma (POAG) have been associated with dysregulation of the outflow of aqueous humor and subsequent elevation of pressure [11]. Oxidative stress on TM cells may lead to DNA damage [12], reduced mitochondrial respiratory activity [13], extracellular matrix accumulation, and release of inflammatory cytokines [14].

Given the importance of oxidative stress in the pathophysiology of glaucoma, the production and presence of free radicals and free radical intermediates in the TM may provide a therapeutic target to restore or prevent further reduction of outflow facility. Toward these ends, ethyl pyruvate (EP) was investigated as a potential therapeutic agent given its known abilities to reduce hydrogen peroxide non-enzymatically [15] and scavenge hydroxyl radicals [16]. To test this hypothesis, EP was assessed for its ability to protect human trabecular meshwork (hTM) cells in culture from peroxide-induced oxidative stress.

The choice of EP as an antioxidative agent is based on EP’s potential to act as an endogenous antioxidant in cells [17]. Mechanistically, this is driven by EP’s ability to reduce hydrogen peroxide non-enzymatically [15,18] and scavenge the reactive oxygen species (ROS) hydroxyl radical [16]. However, the therapeutic utility of pyruvate is limited by its limited solubility in aqueous solutions and its conversion to parapyruvate or pyruvate hydrate, neither of which can scavenge ROS [17]. Thus, EP is the more attractive molecule for therapeutic applications as it is more soluble and stable in aqueous solutions.

## Methods

### Cell culture

Primary hTM cells were sourced from ScienCell (ScienCell Research Laboratories, Carlsbad, CA) [19,20] and used in their third (cytotoxicity testing) and sixth (hydrogen peroxide [H_2_O_2_] stressing) passages. Positive and negative control samples were compared in these two experiments to ensure the difference in passage numbers did not affect the experimental outcomes. The hTM cells were cultured in fibroblast medium (FM; ScienCell) containing 2% fetal bovine serum, 1% fibroblast growth supplement, and 1% penicillin/streptomycin solution (ScienCell). Cells were incubated at 37 °C in a humidified environment with 5% CO_2_. Cells were plated onto collagen-coated 96-well plates at a seeding density of approximately 3×10^3^ cells per well and grown to 75% confluence.

### Cytotoxicity testing

Ethyl pyruvate (Sigma Aldrich, St. Louis, MO) stock solution was prepared in phosphate buffered saline (PBS; 1.06 mM KH_2_PO_4_, 155.17 mM NaCl, 2.97 mM Na_2_HPO_4_-7H_2_O, pH 7.4), and subsequent serial dilutions were prepared in FM medium. Cells were exposed to concentrations of EP ranging from 1 to 20 mM in triplicate. Cells were assayed immediately after EP exposure and at 1, 3, and 5 days after initial exposure. At each time point, an 1-(4,5-dimethylthiazol-2-yl)-2,5-diphenyltetrazolium bromide (MTT) metabolic activity assay and a calcein AM/ethidium homodimer-1 viability/cytotoxicity assay were performed as described below.

### Antioxidant effect

**Figure 1:**
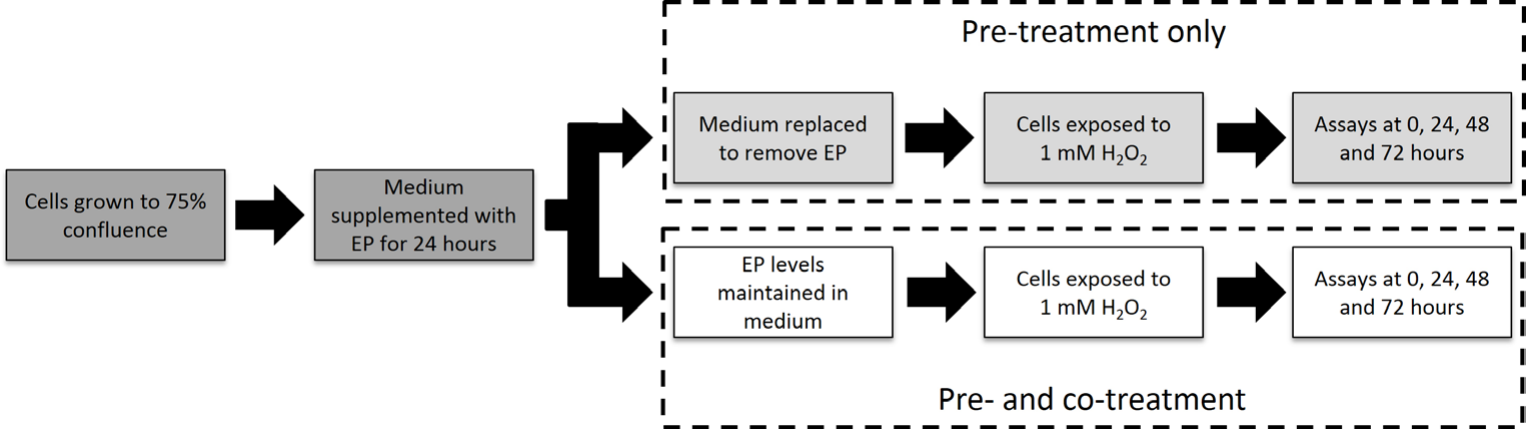
Experimental protocol, EP: ethyl pyruvate. The procedure used to determine the effect of ethyl pyruvate (EP) in protecting cultured human trabecular meshwork cells from peroxide-induced oxidative damage is shown as a diagrammatic representation.

After being plated and grown to 75% confluence, cells were exposed to concentrations of EP ranging from 3 to 10 mM for 24 h. After this initial 24 h exposure, wells were split into two groups: a pretreatment group and a pre- plus cotreatment group. The pretreatment group received the initial 24 h of EP treatment, after which the medium was replaced whereas the pre- plus cotreatment group was maintained in EP-containing medium over the course of the study. After the first 24 h period, the cell culture medium was replaced with medium containing 1 mM H_2_O_2_. This concentration was chosen to give gradual cell death around a level of 50% to provide an adequate signal-to-noise ratio [21]. Live/dead and MTT metabolic activity assays were performed immediately after exposure as well as at 24, 48, and 72 h. This procedure is illustrated diagrammatically in Figure 1.

### 1-(4,5-dimethylthiazol-2-yl)-2,5-diphenyltetrazolium bromide cell metabolic activity assay

Cell metabolic activity was assessed via the MTT assay per the protocol supplied by Invitrogen (Grand Island, NY). Briefly, the medium was removed from each well to be assayed and replaced with 100 μl of fresh medium after which 10 μl of 12 mM MTT stock solution was added to each well. After 4 h incubation at 37 °C, all but 25 μl of medium was removed from the wells and 50 μl of anhydrous DMSO was added to each well and mixed thoroughly. The plates were incubated at 37 °C for 10 min, mixed again, and loaded into a fluorescent plate reader (BioTek Synergy 4, BioTek, Winooski, VT) where absorbance was read at 540 nm. Data were normalized to the MTT absorbance of control TM cells (100% live) and reported as the mean±standard deviation of n=3 samples.

### Cell viability assay

Cell viability was assessed via the calcein AM (CAM) and ethidium homodimer-1 (EthD-1) dye assay per the protocol supplied by Invitrogen. Briefly, cells were rinsed with PBS, and 100 μl of Dulbecco’s PBS (D-PBS, Invitrogen) was added to each well in addition to 100 μl of the combined CAM and EthD-1 reagents (at 2 μM CAM and 4 μM EthD-1). After 15 min incubation at 37 °C, fluorescence readings were taken at 494 nm excitation and 517 nm emission for CAM and 528 nm excitation and 617 nm emission for EthD-1. The CAM and EthD-1 assay data were normalized against samples assayed at 0 h. Data for each treatment are reported as the mean±standard deviation of n=3 samples.

### Data and statistical analysis

Analysis of variance (ANOVA) tests were performed in MATLAB (The MathWorks, Natick, MA) and were used to test for differences in cell metabolic activity and viability between different EP concentrations at each time point throughout the experiment. A significance level of 0.05 was used in all tests. All data are plotted as the mean±standard deviation of three experimental samples.

## Results

### Lower concentrations of ethyl pyruvate are tolerated by cultured human trabecular meshwork cells

**Figure 2:**
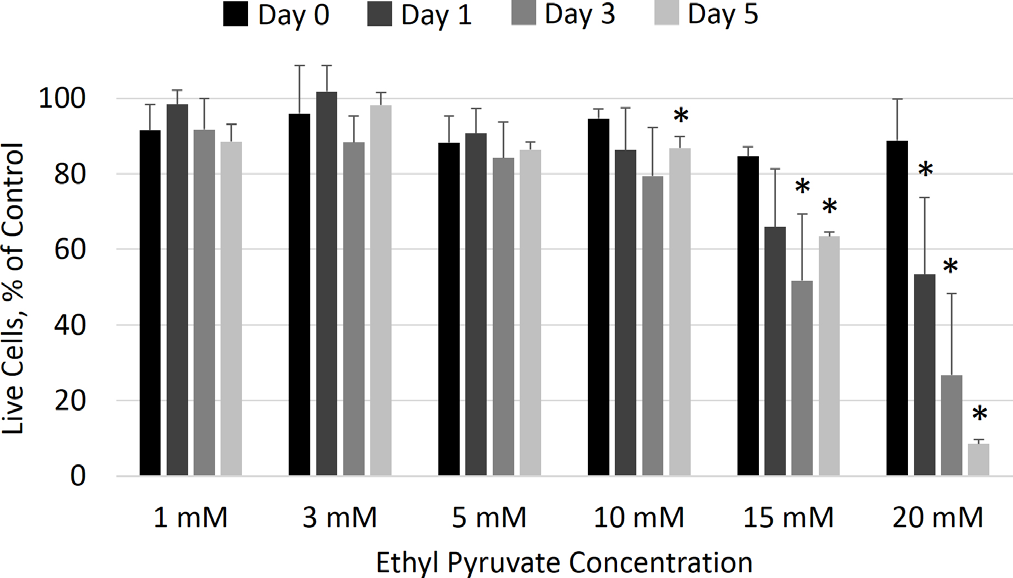
Concentration-dependent toxicity of ethyl pyruvate. The toxicity of various ethyl pyruvate (EP) concentrations was assessed on cultured human trabecular meshwork cells by calcein AM (CAM) staining. Concentrations up to 10 mM showed no toxicity while the 15 and 20 mM samples had fewer live cells at 1, 3, and 5 days. Samples were normalized to 0 mM EP (not shown). The asterisk indicates a statistically significant difference in mean from the day 0 sample at that concentration (p<0.05). Means ± standard deviations for n=3 samples are plotted.

An initial screening study was used to identify candidate concentrations of EP that were not cytotoxic to cultured hTM cells. Shown in Figure 2, these data indicated that concentrations of EP up to 5 mM did not significantly decrease the percentage of live cells remaining. However, the 10, 15, and 20 mM samples showed some levels of cytotoxicity. At 10 mM, only a small decrease in the proportion of live cells was seen after 5 days. The 15 mM condition had significantly fewer live cells at 3 and 5 days compared to the initial sample while the 20 mM condition had fewer live cells at the 1, 3, and 5 day samples. In the 20 mM sample, the percentage of live cells decreased as a function of exposure time. The data shown in Figure 2 were collected using CAM; the EthD-1 and MTT assay data corroborated these results (data not shown).

### Pre- plus cotreated cells are protected from oxidative damage

**Figure 3:**
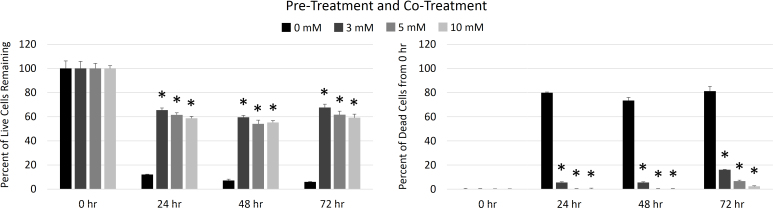
Ethyl pyruvate toxicity by pretreatment and cotreatment. The numbers of cells that remained alive at each experimental time point are expressed as a percent of cells that were alive at 0 h (left panel) and cumulative cell death at each time point is expressed as percent increase in dead cells compared to 0 h samples (right panel). Live and dead cell numbers were determined with calcien AM and ethidium homodimer staining, respectively. Samples that received pre- and cotreatment with ethyl pyruvate (EP) showed markedly increased survival and decreased cell death over control samples. The asterisk indicates a statistically significant difference in the mean from the 0 mM EP samples at that time point (p<0.05). Means ± standard deviations for n=3 samples are plotted.

Following either pretreatment with EP or pretreatment and cotreatment with EP, the hTM cells were stressed with hydrogen peroxide. Live/dead viability and MTT metabolic activity assays were used to quantify oxidative damage to cultured hTM cells and the impact of EP treatment in protecting against such damage. Shown in Figure 3, the percentage of live cells remaining in the pre- and cotreatment group was markedly higher for the samples incubated with EP than the control samples (H_2_O_2_ stressing without EP treatment) but did not correlate with EP concentration. Similarly, in these samples, the percentages of dead cells were lower for the EP-treated samples than the control group, again with no correlation to EP concentration. Even in these samples, some oxidative damage occurred as the 24, 48, and 72 h samples had lower live cell numbers than the initial samples.

**Figure 4:**
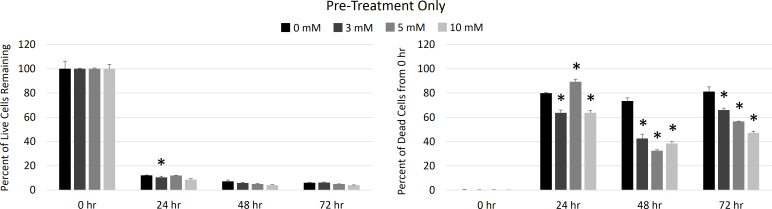
Ethyl pyruvate toxicity by pretreatment only. The numbers of cells that remained alive at each experimental time point are expressed as a percent of cells that were alive at 0 h (left panel) and cumulative cell death at each time point is expressed as percent increase in dead cells compared to 0 h samples (right panel). Live and dead cell numbers were determined with calcien AM and ethidium homodimer staining, respectively. Samples that received only pretreatment with ethyl pyruvate (EP) showed no increase in cell survival and minimal decrease in cell death, compared to control samples. The asterisk indicates a statistically significant difference in the mean from the 0 mM EP samples at that time point (p<0.05). Means ± standard deviations for n=3 samples are plotted.

However, the pretreatment-only group did not show any statistically significant increase in live cells remaining compared to the control samples at any of the EP concentrations tested, as shown in Figure 4. Less than 20% of cells were still alive in all samples after only 24 h of H_2_O_2_ exposure. Although the 48 and 72 h samples showed statistically lower cell death, this difference was negligible when compared to the overall increase in cell death compared to the initial samples.

**Figure 5:**
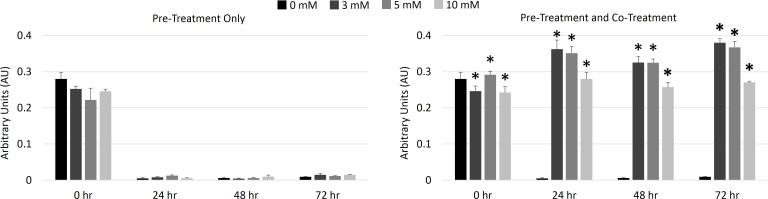
Effect of ethyl pyruvate on metabolic activity. Cell metabolic activity was assessed after 1 mM H_2_O_2_ treatment by 1-(4,5-dimethylthiazol-2-yl)-2,5-diphenyltetrazolium bromide (MTT) assay. Samples that received only pretreatment with ethyl pyruvate (EP) showed nearly no metabolic activity at all time points after initial stressing. Those that received pre- and cotreatment had significantly higher metabolic activity than control samples although this effect was not correlated to EP concentration. The asterisk indicates a statistically significant difference in the mean from the 0 mM EP samples at that time point (p<0.05). Means ± standard deviations for n=3 samples are plotted.

In addition to the live/dead assays, an MTT assay was used to quantify cell metabolic activity. Shown in Figure 5, these data indicate similar results as in the live/dead assay. Samples that only received pretreatment with EP had no statistical differences in metabolic activity compared to control samples that did not receive any EP treatment. However, samples that received pre- and cotreatment with EP had metabolic activity similar to the prestress values at all time points. At the 24, 48, and 72 h time points, the 10 mM EP samples had statistically lower metabolic activity than the 3 and 5 mM samples. Note that since cells were grown to near confluency before the experiment began, it is as expected that metabolic activity did not increase over the course of the 72 h.

## Discussion

In this work, EP was investigated in an in vitro setting for potential as a therapeutic agent to protect hTM cells from peroxide-induced oxidative damage. EP has received interest as a neuroprotective agent in the postischemic brain and heart [22]. In ocular applications, use of EP has mainly focused on preventing and attenuating oxidative damage to the lens as related to cataract formation [23-25] and corneal wound healing [26]. EP’s potential to attenuate oxidative stress effects on TM cells could make it an attractive candidate as a therapeutic agent in treating glaucoma. Ample evidence points toward the significant impact of oxidative stress on the outflow facility of aqueous humor through the TM [27,28]. Because oxidative stress results from an imbalance between oxidant production and cellular antioxidant defense, boosting the endogenous antioxidant capabilities of TM cells or reducing the presence of ROS in the TM may help mitigate oxidative damage and restore outflow pathways. As a starting point toward realizing this therapeutic mechanism, this work investigates the in vitro attenuation of oxidative stress in primary hTM cells with EP.

To justify therapeutic use, EP must not only provide exogenous antioxidant effects (e.g., scavenging free radicals in the aqueous humor) but also increase endogenous intracellular antioxidant capacities. The present investigation was designed to isolate the endogenous and exogenous antioxidative effects of EP in hTM cell cultures when exposed to oxidative stress.

In the first experimental group, the hTM cells underwent 24 h preincubation with EP before peroxide stressing as well as coincubation with EP during stressing (this group was called the “pre- and cotreatment” group). These samples provided a view of EP’s combined exogenous and endogenous antioxidant activity. As shown in the results, this experimental group of hTM cells showed increased cell survival, decreased cell death, and maintenance of metabolic activity after oxidative stressing, indicating EP lent significant antioxidant activity to cultured hTM cells. However, of greater interest from a therapeutic perspective is whether this was largely accounted for by exogenous free radical scavenging or enhancement of endogenous antioxidant capacity.

This question was addressed by the second experimental group, in which hTM cells were incubated with EP for 24 h before peroxide stressing and did not receive EP treatment during the stressing period (this group was called the “pretreatment only” group). Because EP was not present in the medium of these samples, any antioxidant capacity would have come from endogenous activity of the hTM cells themselves. By comparing these samples to the control samples that did not receive EP preincubation, an increase in endogenous antioxidant capacity was identified.

As shown in the results, there was no statistically significant increase in cell survival, decrease in cell death, or maintenance of metabolic activity in these samples compared to the control. As these samples isolated endogenous antioxidant activity, the results suggest that EP does not enhance this capacity in hTM cells, and antioxidant activity is purely exogenous. From a therapeutic perspective, this is not favorable as EP would need to be present in the cellular environment at all times to protect against oxidative damage. In addition, this antioxidant activity depends on the EP concentration present and thus would be susceptible to fluctuations as associated with administration regimens.

Despite EP’s potent antioxidant activity, this work suggests that EP’s potential as a therapeutic agent against oxidative damage in the progression of glaucoma is limited by the inability to enhance endogenous antioxidant capacity of hTM cells in culture. However, if therapeutic concentrations could be maintained for an extended period, such as by a controlled drug delivery system, the full potential of EP for ophthalmic diseases could be realized.
